# Lateral septal PACAP signaling regulates stress and anxiety reactions

**DOI:** 10.1038/s41386-026-02409-y

**Published:** 2026-04-21

**Authors:** Veronica Fontebasso, Federico Ferro, Magali Basille-Dugay, David Vaudry, Jens Hannibal, Nicolas Singewald, Karl Ebner

**Affiliations:** 1 Department of Pharmacology and Toxicology, Institute of Pharmacy and Center for Molecular Biosciences Innsbruck, University of Innsbruck, Innsbruck, Austria; 2 Laboratory of Neuroendocrine, Endocrine and Germinal Differentiation and Communication (NorDiC), Inserm UMR 1239, University Rouen Normandie, Rouen, France; 3 Faculty of Health and Medical Sciences, Institute of Clinical Medicine, University of Copenhagen, Copenhagen, Denmark; 4 Department of Clinical Biochemistry, Faculty of Health Sciences, Bispebjerg and Frederiksberg Hospital, University of Copenhagen, Copenhagen, Denmark

**Keywords:** Stress and resilience, Limbic system

## Abstract

Severe and/or repeated stress exposure can lead to a number of maladaptive physiological and behavioral changes that contribute to psychiatric illnesses. Recent work indicates that the neuropeptide pituitary-adenylate-cyclase-activating-polypeptide (PACAP) plays an important role in stress-related psychopathologies relevant to depression and trauma-related disorders such as PTSD. However, the specific neural circuits that mediate PACAP effects on stress function are not fully understood. One candidate area is the lateral septum (LS), a limbic structure where PACAP and its cognate PAC1 receptors are abundantly expressed. Despite this neuroanatomical evidence, direct functional data supporting a role for septal PACAP/PAC1 receptor signaling in stress regulation are lacking. Using quantitative PCR, we show that forced swim stress increases PACAP mRNA expression in several limbic areas, including the LS, bed nucleus of the stria terminalis and basolateral amygdala, while chronic variable mild stress reduced PACAP expression in the LS only. Providing functional evidence of a PACAP/stress interaction, local administration of PACAP38 into the LS potentiated stress-induced ACTH release and altered stress-coping behavior by increasing passive (floating) and reducing active (struggling) coping during a forced swim challenge. Moreover, intraseptal PACAP38 administration significantly increased anxiety-like behavior in the elevated plus-maze and reduced grooming behavior in the sucrose splashtest, indicating anxiogenic and motivationally disruptive effects following enhanced PACAP signaling in the LS. Importantly, to assess the contribution of endogenous PACAP signaling, intra-LS administration of the PACAP receptor antagonist PACAP(6-38) produced a robust anxiolytic phenotype in the elevated plus-maze. Collectively, these findings provide the first direct evidence that PACAP/PAC1 receptor signaling in the LS modulates emotional and motivational processes in response to stress, identifying this system as a potential target for neuromodulatory interventions in stress-related psychiatric disorders.

## Introduction

Pituitary adenylate cyclase-activating polypeptide (PACAP) is a neuropeptide belonging to the structurally related family of peptides that includes vasoactive intestinal peptide (VIP), glucagon, glucagon-like peptides and secretin [1, 2]. PACAP is the most evolutionarily conserved member of these peptides and is widely expressed in the mammalian brain [3–8]. Encoded by the *ADCYAP1* gene, PACAP is initially translated as a precursor protein that is subsequently processed into two α-amidated active forms, PACAP27 and PACAP38, of which PACAP38 is the predominant isoform in the brain [9–11]. PACAP mediates its effects via three G-protein coupled receptors: the PACAP-preferring PAC1 receptor, which exhibits ~100–1000-fold higher affinity for PACAP than for VIP, and the VPAC1 and VPAC2 receptors, which are shared with VIP and bind both peptides with comparable high affinity [7, 12–14]. The central PACAP/PAC1 receptor system has been implicated in regulating numerous physiological and behavioral functions, including stress and anxiety responses [15–22]. In humans, genetic variants in *ADCYAP1* and *ADCYAP1R1* have been associated with increased susceptibility to psychiatric conditions such as major depression and post-traumatic stress disorder (PTSD) [23–28]. Moreover, post-mortem analyses have shown an upregulation of the PACAP/PAC1 receptor system in the brains of psychiatric patients [29, 30], and elevated circulating PACAP levels have been associated with exaggerated fear responses, autonomic dysfunction [31], as well as, in a predominantly female subgroup, impaired retention of fear extinction alongside intrusive hypervigilant PTSD symptoms [32]. Circulating PACAP levels have also been associated with alterations in brain structure and function in PTSD, including increased amygdala default mode network connectivity [33] and changes in entorhinal cortex neurite density [34], suggesting that PACAP is related to both physiological fear responses and structural functional characteristics of neural circuits implicated in PTSD risk, symptom expression, and arousal-related memory. Although these findings point to a key role for PACAP in psychiatric disorders [35–37], the precise neural mechanisms by which PACAP exerts its effects remain unclear.

Within the stress-regulatory network, PACAP activates the hypothalamic-pituitary-adrenal (HPA) axis through its action on corticotropin-releasing factor (CRF) expressing neurons in the paraventricular nucleus (PVN). Intracerebroventricular administration of PACAP38 increases CRF-mRNA levels and elevates plasma corticosterone in non-stressed animals [38–41]. More recently, we demonstrated that within the PVN, PACAP engages PAC1 receptors on stress-activated CRF neurons to stimulate neuroendocrine stress responses [42]. Conversely, mice lacking PACAP or PAC1 receptors show blunted corticosterone responses to emotional, but not physiological, stressors, indicating a stressor-specific facilitatory role for PACAP in HPA axis activation [21, 43–47]. In addition, PACAP or PAC1 receptor-deficient mice exhibit reduced anxiety-like and fear-related behaviors, along with increased locomotor activity [48–52]. Region-specific manipulations have further identified PACAPergic circuits linking the prefrontal cortex and parabrachial nucleus to the hypothalamus and amygdala, which independently regulate behavioral and endocrine components of the stress response [43]. Thus, beyond its hypothalamic actions, PACAP is also highly expressed in extra-hypothalamic limbic structures, including the bed nucleus of the stria terminalis (BNST) and central amygdala (CeA) [53–56], where PACAP signaling has been strongly implicated in stress-related processing. An emerging structure of particular interest within this network is the lateral septum (LS), which exhibits high PAC1 receptor expression and dense PACAP-immunoreactive innervation [8, 53, 56]. The LS serves as an integrative hub linking hippocampal, hypothalamic, and limbic information to modulate affective, motivational, and neuroendocrine processes [57–61]. Despite this strategic connectivity, the role of PACAP signaling in LS function has received little direct attention. This gap is particularly important given our recent finding that forced swim stress robustly activates PACAP-associated neurons, as indicated by c-Fos expression, in the ventral LS [42]. Therefore, the present study aimed to determine the functional significance of PACAP/PAC1 receptor signaling in the LS in stress and anxiety regulation. Specifically, we examined (1) whether acute or chronic stress exposure alters PACAP and PAC1 receptor mRNA expression in the LS and related limbic regions (e.g., CeA, BNST), and (2) whether local administration of PACAP38 into the LS modulates stress-coping behavior, anxiety-like responses, and neuroendocrine activation. To this end, we evaluated behavioral responses during forced swimming, in the elevated plus-maze, and sucrose splash test (an assay particularly sensitive to motivational and anhedonia-like deficits [62, 63]). We hypothesized that stress exposure enhances PACAP signaling in the LS and that local activation of LS PACAP/PAC1 receptors facilitates HPA axis responses, changes stress-coping and motivational behavior and, increases anxiety.

## Material and methods

Detailed materials and methods can be found in the Supplementary Material.

### Animals

All experimental procedures on adult male Sprague-Dawley rats were approved by the national Ethical Committee on animal care and use in compliance with international laws and policies.

### Surgery

All surgical procedures were performed under sterile conditions as previously described [42]. Guide cannulas for intracerebral microinfusions were implanted either unilaterally 1 mm above the right lateral ventricle (coordinates from bregma: AP 0.8 mm caudal, ML + 1.4 mm and DV −3.0 mm) or bilaterally 2 mm above the LS (coordinates from bregma: AP 0.6 mm rostral, ML+/− 1.7 mm, DV −4.0 mm, with an angle of 10°) or rostral portion of the anterolateral BNST (coordinates from bregma: AP 0.2 mm rostral, ML +/− 1.8 mm, DV −5.0 mm) by using a rat brain atlas [64]. During recovery single-housed rats received analgesic care and the locations of the cannula track were histologically verified after experiments (see Supplementary Fig. S1).

#### Implantation of a jugular venous catheter

A silastic-tipped vinyl catheter was inserted into the left jugular vein, routed under the skin and exteriorized at the neck of the animal as described previously [42]. Blood sampling through a pre-implanted jugular venous catheter allows repeated blood sampling from conscious, freely-moving rats without restraining animals.

### Behavioral procedures

#### Forced swim challenge

The forced swim challenge was conducted as described previously [42].

#### Elevated plus-maze test

The elevated plus maze consisted of two open and two closed arms elevated 75 cm above the floor, and rats were placed in the center and allowed to explore for 5 min. Behavior was recorded and analyzed using automated video tracking to quantify open-arm exploration and overall activity.

#### Sucrose splash test

The splash test assessed motivational and self-care behavior by spraying a 10% sucrose solution onto the rat’s dorsal coat and recording grooming for 15 min. Grooming duration, number of bouts, and latency to the first bout were manually scored by a blinded observer.

### Stress paradigms for qPCR analysis

#### Acute swim stress

Rats underwent a two-day forced swim procedure in 20 °C water (5 min on day 1, 10 min on day 2) as described previously [65].

#### Chronic variable mild stress

Rats were subjected to a 7-day chronic variable mild stress paradigm, as previously described [65]. Each day, animals experienced one mild stressor presented in a randomized and unpredictable order, with certain stressors repeated across the week to increase unpredictability. The stressors were chosen to be mild yet effective in inducing a sustained stress response (Supplementary Table S1). Two hours after the final stress session, rats were euthanized, and their brains were rapidly extracted, snap-frozen in isopentane (Sigma-Aldrich, Austria) chilled on dry ice, and subsequently stored at −80 °C until further PCR processing.

### Drug microinjection procedure

For bilateral microinfusions into the LS or anterolateral BNST with simultaneous blood sampling we used a rather ‘stress-free’ drug administration approach as described previously [42]. Briefly, stylets of guide cannulas were replaced by two 30-gauge microinjection cannulas that were 2 mm longer than the guide cannulas, thus reaching the LS or BNST. Injection cannulas were connected to a 5 cm long PE-10 tubing and filled with drug or vehicle solution. This infusion device was connected to a syringe mounted on a microinfusion pump (TSE-Systems, Bad Homburg, Germany) via a 100 cm long polyethylene tubing interconnected with a dual channel fluid swivel system (Instec Laboratories) and mounted at least 1 h for starting blood sampling. Drugs were infused over a period of 7.5 min at a defined flow rate of 0.2 µl/min (1.5 µl/injection side). After the microinjection procedure, the injection cannula was left in the guide cannula for an additional 2.5 min before being removed. Thereafter, animals were exposed to the forced swim challenge.

For intracerebroventricular or intraseptal microinfusions without concurrent blood sampling, rats were gently restrained in their home cages and stylets were removed. Drugs were injected into the lateral ventricle or LS (1.5 µl/site) by manual infusion over a period of ~7.5 min using a 5-µl Hamilton syringe (Hamilton Instruments, Switzerland) connected via polyethylene tubing to a 30-gauge injection cannula (tip extending 1–2 mm beyond the guide). Following infusion, the internal cannula was left in place for an additional 2 min before withdrawal. Behavioral testing in the elevated plus-maze or splash test followed 15 min later.

### Blood sampling and ACTH measurements

After a 60-min habituation, two basal blood samples were collected 35 and 15 min before stress exposure. Drugs were infused into the LS or anterolateral BNST starting 12 min before the stressor, followed by another blood sample and a 5-min forced swim challenge. Three additional blood samples were collected 10, 30, and 60 min after stressor onset, with each sample volume replaced by heparinized saline. Plasma was separated and stored at −80 °C, and ACTH levels were measured using a commercial immunoassay kit with assay variability below 10%.

### Drugs

PACAP38 and PACAP(6-38) were dissolved in sterile distilled water and aliquots of stock solution (1 mg/mL) were stored at −80 °C. For preparation of the working solution the liquid of concentrated stock solution was diluted with artificial cerebrospinal fluid (aCSF) to a final concentration of 10 or 100 µM. Vehicle animals received infusions of aCSF solution. All drugs were freshly prepared before each experiment and kept on ice during the experimental procedures.

### Brain punching and quantitative real-time PCR

The method of tissue dissection, RNA extraction and quantitative reverse transcription-PCR procedure using specific primers (Supplementary Table S2) was conducted as described previously [65]. Bilateral tissue punches from the LS, PVN, BNST, CeA, basolateral amygdala (BLA) and medial amygdala (MeA) were collected.

### Immunofluorescent histochemistry

Immunofluorescence staining and photomicroscopy was performed as previously described [42, 66].

### Statistics

Data are expressed as mean ± standard error of the mean (SEM). Statistical analysis of data was performed using GraphPad Prism 10 (GraphPad software Inc, USA). Statistical significance was accepted if *p* < 0.05.

## Results

### Acute or chronic stress alters PACAP/PAC1 receptor mRNA levels within different limbic brain areas

**Fig. 1:**
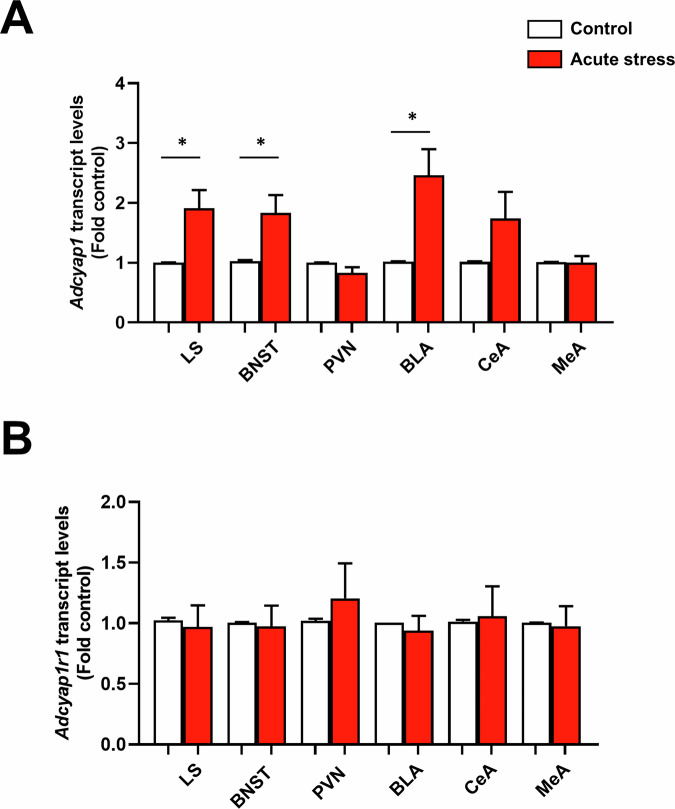
Effects of acute swim stress on PACAP and PAC1 mRNA transcript levels in distinct forebrain areas of rats. Adult male rats were exposed to swim stress on two consecutive days and distinct brain regions were harvested for quantitative PCR analyses of **(A)** PACAP (*Adcyap1*) and **(B**) PAC1 receptor (*Adcyap1r1*) mRNA transcription levels. All RNA tissue samples from each region were reverse transcribed at the same time with random hexamers to allow quantitation and normalization across samples against Gapdh, B2m and Actb transcript levels as reference/housekeeping genes. Fold changes in gene expression of acute-stressed animals (red bars; *n* = 5–6) were calculated using the 2^-ΔΔCt^ method and compared to unstressed controls (white bars; *n* = 4). Data are expressed as group means ± SEM. Abbreviations: LS lateral septum, BNST bed nucleus of the stria terminalis, PVN paraventricular nucleus, BLA basolateral amygdala, CeA central amygdala, MeA medial amygdala. *p < 0.05 compared to controls (Mann–Whitney U-test).

In the present study, we examined the effects of acute swim stress or chronic variable mild stress on the PACAP and PAC1 receptor gene expression in selected forebrain areas known to be implicated in stress and anxiety regulation. Because the acute (forced swim) and chronic (seven-day mild variable stress) stress paradigms differ in type and duration and thus cannot be directly compared, they were analyzed as separate conditions. As shown in Fig. 1A, animals exposed to forced swim stress on two consecutive days showed increased PACAP mRNA transcripts levels in the LS (*p* < 0.05), BNST (*p* < 0.05) and BLA (*p* < 0.05) compared to unstressed control animals. In contrast, swim stress did not alter PACAP transcript levels in the other stress-related brain region investigated, including the PVN, CeA and MeA. To determine whether changes of PACAP transcript levels were associated with alterations of receptor expression, the same cDNA template were also analyzed for PAC1 receptor expression. However, PAC1 mRNA levels did not differ from those of non-stressed controls in any brain region examined (Fig. 1B), indicating that swim stress had no acute effect on PAC1 receptor expression.

We next investigated whether chronic stress modulates the expression of the PACAP/PAC1 receptor system within the selected limbic brain areas. Notably, chronic stress, induced by one week of chronic variable mild stress exposure, significantly reduced PACAP transcript levels in the LS by 35% (*p* = 0.0095) compared to unstressed controls (Supplementary Fig. S2A) while PACAP expression remained unchanged in all other regions examined following chronic stress (Supplementary Fig. S2A). Interestingly, rats exposed to chronic stress exhibited a slight but significant increase in PAC1 receptor expression within the BNST (*p* < 0.05) compared to controls (Supplementary Fig. S2B).

### Intra-LS administration of PACAP38 modulates behavioral and neuroendocrine stress response

**Fig. 2:**
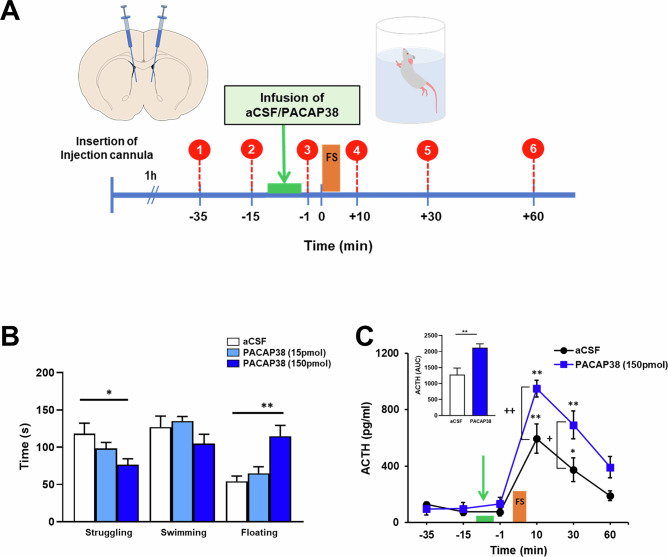
Effects of bilateral microinjection of PACAP38 into the LS on behavioral and neuroendocrine responses to forced swim stress. **(A)** Schematic illustration of the experimental design with the timeline of blood sampling (red circles), drug infusion (green bar) and stress exposure (orange bar, forced swim challenge, FS). Experiment started with insertion of the infusion device (bilateral injection cannulas connected to a microinfusion pump) at least 1 h before blood sampling started. Drugs were infused automatically at a constant flow rate over a period of 7.5 min without any stressful manipulations (e.g such as capturing or restraining animals) before and during the infusion procedure. Blood samples were collected at regular intervals before drug infusion under basal conditions (−35 and −15 min) and after drug infusion, but before stress exposure (−1 min) and after forced swim stress (10, 30, and 60 min). **(B)** Rats infused with different doses of PACAP38 into the LS (15 and 150 pmol/site; *n* = 5 and 7) showed significantly increased floating (passive coping) and decreased struggling (active coping) compared to aCSF-injected controls (*n* = 8), with the effect more pronounced at the higher dose. No significant effects were found on the swimming behavior between PACAP38-injected rats and controls. *p < 0.05, **p < 0.01 compared to vehicle-injected controls (1-way ANOVA followed by Dunnett’s multiple comparison post hoc test). **(C)** Swim stress caused an increase in plasma ACTH levels in both intra-LS PACAP38 (*n* = 6) and vehicle-injected controls (*n* = 6). Compared to controls, intra-LS PACAP38-injected rats showed higher plasma ACTH levels during and after forced swim stress. However, basal levels did not differ between groups. The green bar indicates timing of intra-LS infusion, the orange bar the forced swim (FS) stress exposure. *p < 0.05, **p < 0.01 compared to basal timepoints (−35 and −15 min) in same treatment group; ++*p* < 0.01, +*p* < 0.05 compared to vehicle-injected controls at same timepoint (2-way ANOVA followed by Bonferroni’s multiple comparison post hoc test). The inset shows the area under the curve (AUC) quantification of ACTH levels, which was significantly higher in PACAP38-injected animals than in vehicle-treated controls (**p < 0.01, Student’s *t* test). Data are expressed as mean ± SEM.

Histological analysis confirmed that intra-LS injections were located between 1.0 and 0.2 mm anterior to bregma. Only animals with both cannulas correctly placed in the LS (Supplementary Fig. S1) and functioning properly were included in this study. Of the 22 rats implanted with guide cannulas, two animals were excluded from further experiments due to cannula occlusion. To investigate the role of PACAP signaling in the LS during stress, PACAP38 was locally administered into the LS (Fig. 2A) of male rats at two doses (15 or 150 pmol; *n* = 5 and 7), and stress-coping behavior during forced swimming was compared to that of control animals receiving vehicle (aCSF; *n* = 8). As shown in Fig. 2B, intra-septal PACAP38 administration significantly increased passive coping (floating) and reduced active coping (struggling) behaviors in a dose-dependent manner. Statistical analysis by one-way ANOVA revealed a significant effect of treatment on floating behavior (*F*(2,17) = 8.51, *p* = 0.0027). Post hoc analysis indicated that the higher dose (150 pmol) of PACAP38 significantly increased floating time compared to vehicle-treated controls (*p* < 0.01), while the lower dose (15 pmol) was less effective and did not reach significance. In addition, struggling behavior was also significantly affected by treatment (*F*(2,17) = 3.769, *p* = 0.0442). Post hoc comparisons revealed that the higher dose of PACAP38 significantly reduced struggling time compared to the control group (*p* < 0.05) while the administration of the lower dose of PACAP38 showed a non-significant trend toward reduced struggling behavior. No significant differences were found in swimming behavior across treatment groups (*F*(2,17) = 1.135, *p* = 0.2841).

Next, we examined whether enhanced septal PACAP signaling also modulates measures of HPA axis activity. To this end, plasma ACTH levels were measured before and at several time points following forced swimming (Fig. 2A) in rats that received intra-LS injections of either PACAP38 (150 pmol/site) or vehicle (aCSF). Three animals were excluded from the analysis due to catheter blockages that prevented blood sampling during the experiment, resulting in a final *n* = 6 per group (controls and PACAP38, 150 pmol). As shown in Fig. 2C, swim stress induced an increase in plasma ACTH levels in both treatment groups. Two-way ANOVA revealed significant main effects of treatment (*F*(1,10) = 12.18, *p* = 0.0058) and time (*F*(5,50) = 46.86, *p* < 0.0001), as well as a significant treatment x time interaction (*F*(5,50) = 3.854 *p* = 0.005). Subsequent Bonferroni´s post hoc analysis indicated that PACAP38-injected rats displayed significantly higher plasma ACTH levels at 10 (*p* < 0.01) and 30 min (*p* < 0.05) after the onset of swim stress, compared to aCSF-injected controls. Consistent with this time-course analysis, area under the curve (AUC) quantification revealed a significantly greater overall ACTH response in PACAP38-injected rats compared to controls (*t*(10) = 3.466, *p* = 0.0061; Student’s *t* test; Fig. 2C, inset). In contrast, baseline ACTH levels did not differ between groups, suggesting that PACAP38 selectively enhances the stress-induced activation of the HPA axis without altering basal hormone secretion. In a separate cohort of animals, we measured ACTH plasma levels in control animals without stress exposure over the same entire time course of the experiment following intra-LS PACAP38 administration. Our findings show that ACTH levels did not significantly differ between PACAP38-treated animals (*n* = 3) and controls (*n* = 4) at any point during the experiment, indicating that PACAP38 did not induce a sustained rise in ACTH levels in the absence of stress (Supplementary Fig. S3).

### Intra-BNST administration of PACAP38 does not alter behavioral and neuroendocrine stress response

To assess whether LS microinjections potentially affected adjacent brain regions, we directly infused PACAP38 into the rostral anterolateral BNST (Supplementary Fig. S4), an area that has also shown increased PACAP levels following acute stress. Male rats received intra-BNST PACAP38 (150 pmol) or aCSF (control) and were evaluated during the forced swim test. PACAP38 produced no significant changes in any behavioral measure, as Student´s t-test revealed no statistical significance neither in struggling (*t*(14) = 0.673, *p* = 0.512), swimming (*t*(14) = 1.319, *p* = 0.208), or floating behavior (*t*(14) = 0.216, *p* = 0.832) (Supplementary Fig. S4A). Consistent with the behavioral data, PACAP38 did not alter HPA axis activity, as neither basal nor stress-induced plasma ACTH levels differed between groups (Supplementary Fig. S4B).

### Intra-LS administration of PACAP38 increases anxiety-related behavior

**Fig. 3:**
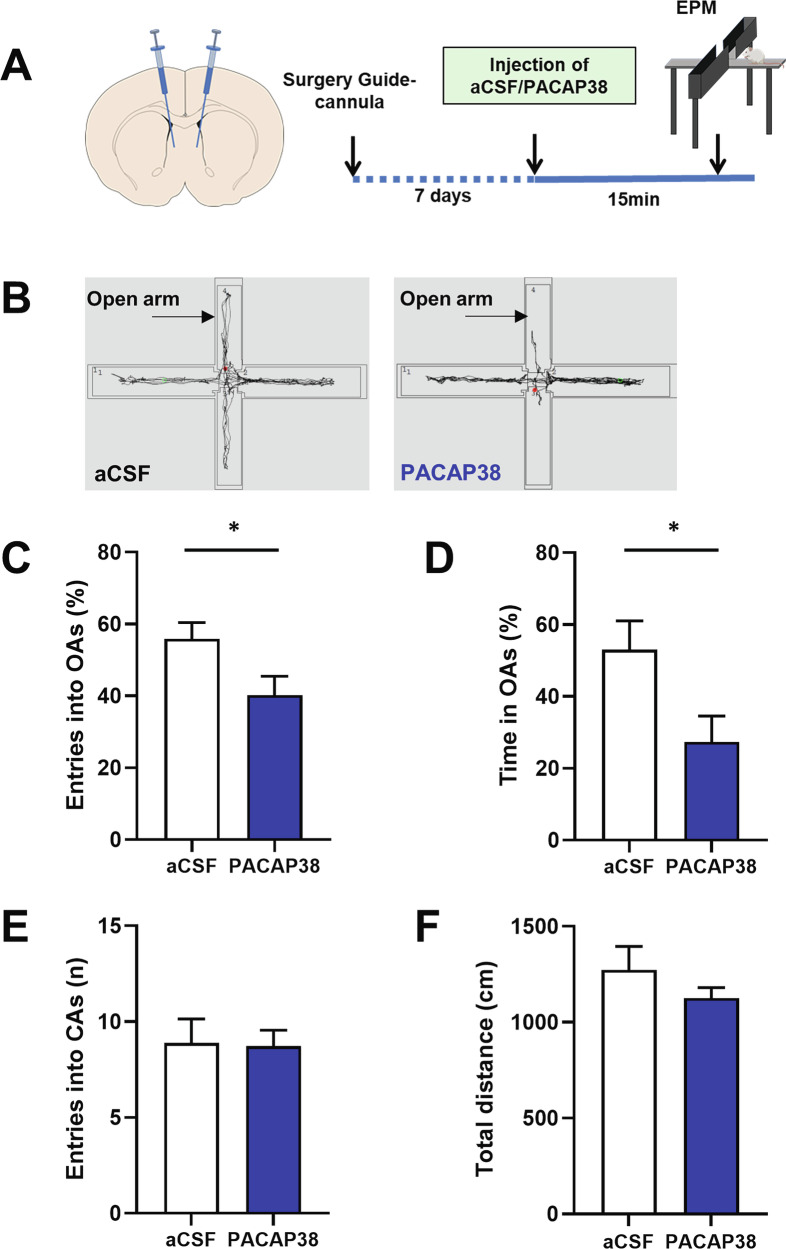
Effects of PACAP38 administration into the LS on the anxiety-related behavior of rats tested in the elevated plus-maze test. **(A)** Schematic illustration of the experimental design with the timeline of surgery, drug infusion and behavioral testing. **(B)** Representative examples of motion-tracking images on the plus maze of a vehicle (left panel) and PACAP38 (right panel) injected rat. Results revealed that PACAP38 (150 pmol/site) treated rats showed reduced open-arm exploration, consistent with an anxiogenic-like effect, indicated by a significant decrease of the percentage of entries into open arms (**C**) and in the percentage of time spent in the open arms (**D**) compared to vehicle-injected controls. There was no significant difference in the number of entries into closed arms (**E**) or in the total distance traveled (**F**) between groups. Abbreviations: EPM elevated plus-maze, CAs closed arms, OAs open arms. Data are expressed as mean ± SEM, *n* = 7–9 per group. *p < 0.05 compared to vehicle-injected controls (Student’s *t* test).

To examine the role of septal PACAP signaling in anxiety-like behavior, a separate cohort of male rats received bilateral intra-LS microinjections of PACAP38 (150 pmol; *n* = 7) or vehicle (aCSF; *n* = 9) and were subsequently tested on the elevated plus-maze (Fig. 3A). As shown in Fig. 3B–D, PACAP38 administration significantly reduced open-arm exploration, consistent with an anxiogenic-like effect. Specifically, unpaired Student’s t-tests revealed that PACAP38-treated rats exhibited a nearly 30% reduction in the percentage of open-arm entries (*t*(14) = 2.264, *p* = 0.040; Fig. 3C) and a 50% decrease in the percentage of time spent in the open arms (*t*(14) = 2.297, *p* = 0.037; Fig. 3D), compared to vehicle-treated controls. Importantly, no significant group differences were observed in the total distance traveled (*t*(14) = 0.990, *p* = 0.338; Fig. 3F) or in the number of closed-arm entries (*t*(14) = 0.108, *p* = 0.915; Fig. 3E), indicating that the observed effects were specific to anxiety-related behavior and not attributable to changes in locomotor activity.

### Intraseptal administration of PACAP(6-38) reduces anxiety-related behavior

**Fig. 4:**
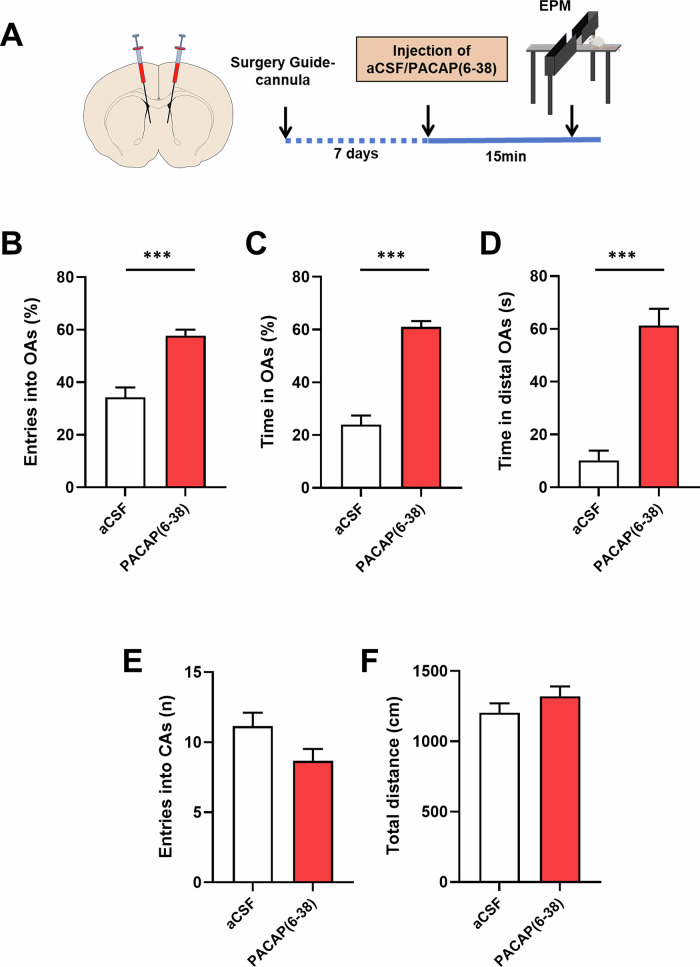
Effects of PACAP(6-38) administration into the LS on the anxiety-related behavior of rats tested in the elevated plus-maze test. **(A)** Schematic illustration of the experimental design with the timeline of surgery, drug infusion, and behavioral testing. **(B)** Intra-LS administration of PACAP(6-38) (150 pmol/site) significantly increased the proportion of entries into the open arms compared to vehicle-treated animals. **(C)** Similarly, PACAP(6-38)-treated rats spent a greater percentage of time in the open arms, and **(D**) analysis of open-arm subregions revealed that PACAP(6-38)-treated animals spent more time in the distal portions of the open arms, further supporting an anxiolytic-like effect. No differences were detected in closed-arm entries (**E**), or in the total distance traveled (**F**) between groups, indicating that general locomotor activity was not altered by the treatment. Abbreviations: EPM elevated plus-maze, CAs closed arms, OAs open arms. Data are expressed as mean ± SEM, *n* = 7–9 per group. ***p < 0.001 compared to vehicle-treated controls (Student’s *t* test).

To assess the contribution of endogenous PACAP signaling within the LS to anxiety-related behavior, a separate cohort of rats was bilaterally infused with aCSF (controls, *n* = 7) or the PACAP receptor antagonist PACAP(6-38) (150 pmol/site, *n* = 9) and tested in the elevated plus-maze (Fig. 4A) following the same experimental procedures as described above. In line with an anxiolytic-like profile, PACAP(6-38) treatment enhanced open-arm exploration (Fig. 4B-D). Statistical analysis using unpaired Student’s t-tests demonstrated that PACAP(6-38)-treated rats showed a 65% increase in the percentage of open-arm entries (*t*(14) = 5.622, *p* < 0.0001; Fig. 4B) and an approximately 150% increase in the percentage of time spent in the open arms (*t*(14) = 9.449, *p* < 0.0001; Fig. 4C), compared to vehicle-treated controls. In addition, PACAP(6-38)-treated animals spent markedly more time in the distal portions of the open arms, representing the most anxiogenic zone of the maze (*t*(14) = 6.471, *p* < 0.0001; Fig. 4D). Importantly, no significant group differences were observed in the total number of closed-arm entries (*t*(14) = 1.929, *p* = 0. 0743; Fig. 4E) or in the total distance traveled (*t*(14) = 1.186, *p* = 0.255; Fig. 4F), indicating that the behavioral effects were not attributable to altered general locomotor activity levels.

### Intra-LS administration of PACAP38 modulates motivational behavior

**Fig. 5:**
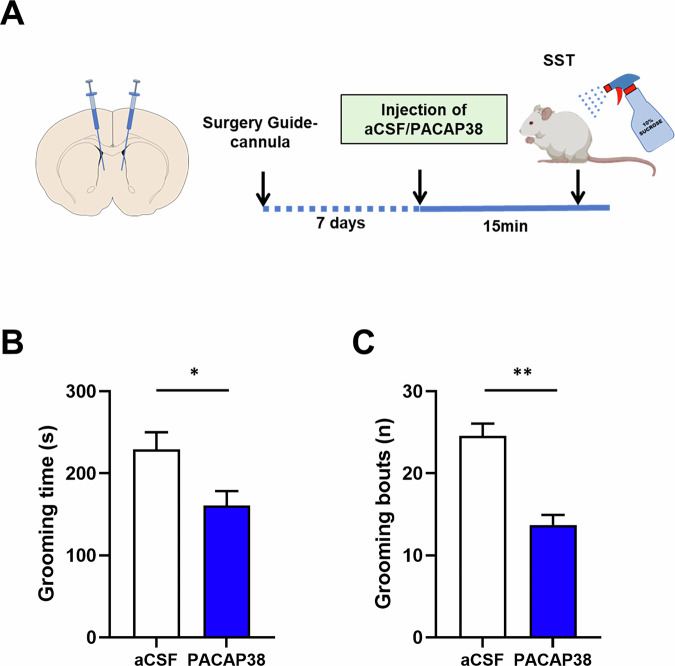
Effects of PACAP38 administration into the LS on the motivational self-care behavior of rats tested in the sucrose splash test. **(A)** Schematic illustration of the experimental design with the timeline of surgery, drug infusion and behavioral testing. Results revealed that PACAP38 (150 pmol/site) treated rats showed a significant reduction of time spent grooming (**B**) and a decrease in the number of grooming bouts (**C**) compared to aCSF-injected control rats. Abbreviations: SST sucrose splash test. Data are expressed as mean ± SEM, *n* = 6 per group. *p < 0.05, **p < 0.01 compared to vehicle-injected controls (Student’s *t* test).

To minimize animal use, the same cohort of rats previously tested in the elevated plus-maze following intra-LS PACAP38 administration was used again one week later for the sucrose splash test (Fig. 5A). This interval is generally considered sufficient for complete washout of pharmacological agents, and each animal received the same treatment during both tests. Of the 16 animals implanted with bilateral guide cannulas into the LS, two were excluded due to cannula blockages, resulting in a final *n* = 7 per group (controls and PACAP38, 150 pmol). The sucrose splash test measures spontaneous grooming behavior as an index of self-care and motivational drive. As shown in Fig. 5, intra-LS administration of PACAP38 (150 pmol) significantly reduced total grooming time compared to vehicle controls (*t*(12) = 2.487, *p* = 0.0286; Fig. 5B) and markedly decreased the number of grooming bouts by almost 50% (*t*(12) = 5.613, *p* = 0.0001; Fig. 5C). In contrast, the latency to first grooming did not differ significantly between groups (*t*(12) = 1.754, *p* = 0.1048; data not shown). These findings suggest that heightened PACAP activity within the LS disrupts self-directed grooming behavior, supporting a role of septal PACAP signaling in motivational and self-care processes.

### Distribution of PAC1 receptors in the LS and co-localization with stress-induced c-Fos expression

**Fig. 6:**
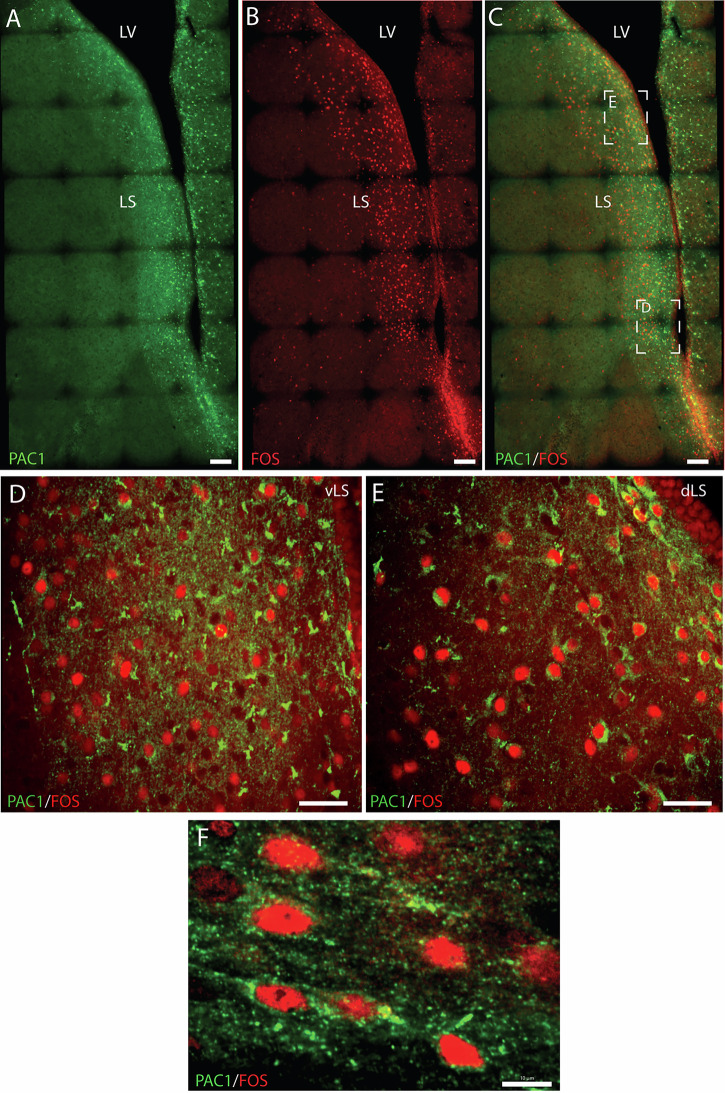
Distribution of PAC1 receptor and c-Fos immunoreactivity in the LS. Representative confocal photomicrographs illustrating dual immunofluorescence labeling of PAC1 receptor (green, **A**) and c-Fos (red, **B**) expression as well as their co-expression (merged image, **C**) in the LS of a rat exposed to forced swim stress. High-magnification images (**D**–**F**) show co-expression of PAC1 and c-Fos in neurons located in both the ventral LS and the dorsal LS, with a slightly higher degree of overlap in the ventral LS than in the dorsal LS. PAC1 immunoreactivity appeared as dot-like staining along the cell membrane and proximal dendrites of most c-Fos-positive neurons (**F**). Abbreviations: LS lateral septum, vLS ventral LS, dLS dorsal LS, LV lateral ventricle. Scale bars: **A**–**C** 100 μm; **D**, **E** 25 μm; **F** 10 µm.

Immunohistochemistry was used to examine the co-localization of PAC1 and c-Fos in the LS of a rat exposed to forced swim stress (Fig. 6). Because c-Fos expression in the LS is minimal under basal, non-stressed conditions [42], assessment of PAC1/c-Fos co-localization is most informative following a stress challenge. Accordingly, all co-localization analyses were performed in tissue from a stressed animal. This animal received an intracerebroventricular PACAP38 infusion prior to the stress session. Notably, c-Fos positive neurons were observed in both the dorsal and ventral LS (Fig. 6B, C), and PAC1-expressing neurons were identifiable throughout the LS (Fig. 6A, D–F). PAC1 immunoreactivity appeared as dot-like staining along the cell membrane and proximal dendrites of many c-Fos expressing neurons (Fig. 6D–F), consistent with a synaptic or perisynaptic localization in stress-activated LS neurons. Qualitatively, the overlap between PAC1 and c-Fos appeared more prominent in the ventral LS than in the dorsal LS. This overlap suggests that neuronal activation in the LS is closely associated with PAC1 receptor expression, with a somewhat stronger coupling in the ventral LS compared to the dorsal LS. In line with this, supplementary data from an unstressed control rat show that PACAP is present in nerve fibers innervating LS neurons, which express PAC1 receptors on their membranes and processes (Supplementary Fig. S5). A higher-magnification image reveals examples of PACAP-positive nerve terminals in close apposition to PAC1 immunoreactivity on the cell membrane, likely corresponding to synaptic contacts (Supplementary Fig. S5B). This observation supports a functional link between PACAP-containing afferents and PAC1 receptor-expressing LS neurons under basal conditions, providing anatomical context for the stress-induced activation observed in Fig. 6.

## Discussion

The present study identifies a critical role for PACAP/PAC1 receptor signaling within the LS in modulating stress and anxiety responses. We first demonstrate that exposure to acute stress (forced swim) increased PACAP transcript levels in the LS, indicating enhanced PACAPergic activity in this region following aversive and stressful stimuli. Functionally, local administration of PACAP38 into the LS induced a shift to maladaptive coping behavior during forced swimming by promoting passive (floating) and reducing active (struggling) coping. This behavioral shift was accompanied by an exaggerated stress-induced release of the stress hormone ACTH, reflecting potentiation of the HPA stress axis. Furthermore, intra-LS PACAP38 administration decreased open arm exploration in the elevated plus-maze and reduced grooming behavior in the sucrose splash test, suggesting anxiogenic-like effects and impaired motivational drive. Importantly, pharmacological blockade of PACAP signaling using PACAP(6-38) within the LS produced an anxiolytic-like effect in the elevated plus-maze, providing direct functional evidence that endogenous PACAP signaling contributes to the regulation of anxiety-related behavior. Immunohistochemistry demonstrated that the majority of stress-activated neurons in the LS expressing c-Fos co-express the PAC1 receptor. Collectively, these findings provide the first direct evidence that PACAP/PAC1 receptor signaling in the LS regulates key aspects of stress-related emotional and motivational behavior, highlighting the septal PACAP system as a potential neuromodulatory target for interventions in stress-related psychiatric conditions such as anxiety disorders and depression.

### Stress-related molecular regulation of PACAP and PAC1 receptors in limbic regions

Our first aim was to examine whether the exposure to emotionally salient stressors modulates the PACAP/PAC1 receptor system in limbic brain regions. Using quantitative PCR, we found that repeated forced swim stress on two consecutive days significantly increased PACAP mRNA expression in the LS, BNST and BLA. A non-significant trend toward increased PACAP expression was observed in the CeA, whereas no changes were detected in the PVN. Notably, PAC1 receptor mRNA levels remained unchanged across all examined brain regions following acute swim stress. In contrast, exposure to a chronic variable mild stress paradigm over seven days produced a distinct expression profile, with a downregulation of PACAP mRNA in the LS and an upregulation of PAC1 mRNA in the BNST. Hence, these findings provide novel insights into the dynamic regulation of PACAP signaling across stress paradigms of varying duration and nature. The increase of PACAP mRNA following swim stress, a notably potent but acute challenge [60, 67], suggests that PACAPergic transmission in limbic structures such as the LS, BNST, and BLA is particularly sensitive to emotionally salient, short-term stressors. This observation aligns with earlier immunohistochemical studies reporting augmented PACAP peptide density in the BNST and amygdala after acute stressors such as footshock or single social defeat [68, 69]. Importantly, our results extend this work by showing that while acute swim stress did not alter PAC1 receptor expression, chronic mild stress selectively induced PAC1 upregulation in the BNST. This suggests that chronic and variable stressors may be required to drive PAC1 plasticity within specific limbic circuits. These results are partially consistent with previous work by Hammack and colleagues, who reported that chronic variate stress robustly increases PACAP and PAC1 mRNA expression in the BNST [70, 71]. Specifically, they observed a two-fold increase in PAC1 receptor mRNA together with a striking 10-fold increase in PACAP transcript selectively within the dorsal BNST after 7 days of chronic variate stress, with no significant changes in other limbic regions such as ventral BNST, amygdala or LS [70]. In contrast, we found no upregulation of PACAP mRNA in the BNST, and instead detected a significant 35% downregulation of PACAP transcript levels in the LS after chronic stress. Several factors may account for this divergence, including differences in stressor modalities, timing of tissue collection (2 h post-stress in our study vs. 24 h in Hammack’s study), or subtle anatomical sampling variations. Notably, Hammack and colleagues used highly localized micropunches restricted to the dorsal aspect of the anterolateral BNST, whereas our analysis pooled tissue from both dorsal and ventral BNST. Such methodological differences could dilute subregion-specific effects and mask localized PACAP upregulation within the dorsal BNST. Given the well-established heterogeneity of BNST subnuclei, even minor variation in micropunch placement or dissection boundaries may substantially affect the detection of stress-induced molecular changes. Importantly, while we did not replicate this BNST-specific upregulation, we identified a robust and regionally selective downregulation of PACAP in the LS. The opposing regulation of LS PACAP across acute versus chronic stress exposure is particularly noteworthy. This bidirectional pattern suggests that septal PACAP signaling is dynamically regulated, potentially mobilized during acute threat to facilitate adaptive responses, but suppressed under prolonged stress, perhaps as a compensatory or maladaptive response mechanism. Such modulation is consistent with the proposed role of the LS in coordinating physiological and behavioral stress responses [59, 60]. Moreover, given that the LS itself comprises distinct subregions (dorsal, intermediate, and ventral), future work should aim to resolve potential subregion-specific regulation of PACAP under different stress conditions.

### Behavioral and neuroendocrine effects of septal PACAP signaling

To further assess a functional role of septal PACAP signaling in stress regulation, we examined the effects of local PACAP administration into the LS on behavioral and neuroendocrine stress responses. Intra-LS infusions of PACAP38 significantly increased floating time and reduced struggling during forced swimming, indicating a shift toward passive coping strategies when facing inescapable stress. This behavioral change was accompanied by an exaggerated HPA axis stress response. Specifically, intra-septal PACAP38 administration potentiated the swim stress-induced ACTH increase without altering basal ACTH levels, suggesting a selective amplification of stress reactivity rather than baseline activity. Together, these findings provide direct functional evidence that PACAP signaling in the LS modulates both behavioral and neuroendocrine responses to stress. Importantly, the behavioral and endocrine effects observed after intra-LS PACAP administration are unlikely to reflect diffusion into neighboring structures. Infusions directed at the rostral anterolateral BNST, an area anatomically adjacent to the LS, did not produce significant changes in stress-coping behavior or ACTH release. This indicates that the observed effects arise predominantly from PACAP actions within the LS rather than from spillover into nearby regions and supports the conclusion that LS-specific PACAP signaling drives the behavioral and neuroendocrine outcomes identified in this study. Nevertheless, as with all intracerebral microinjection approaches, some degree of off-target diffusion cannot be completely excluded. Importantly, the conclusion of LS-specific action is further supported by our previous observations using intracerebroventricular PACAP administration, where increased passive coping was accompanied by heightened swim stress-induced neuronal activity in the LS, as indicated by c-Fos expression [42]. Notably, our brain mapping studies revealed that PACAP enhanced stress-induced c-Fos expression in the ventral part of the LS, a region strongly activated by various stressors, including forced swim [72–74], and is implicated in the regulation of passive and fear-related responses to stress [59, 75]. In the present study, we further observed that swim stress activates PAC1 receptor-positive neurons not only in the ventral LS but also in the intermediate and dorsal part (see Fig. 6), suggesting that PACAP-induced stress effects are likely mediated through multiple LS subdivisions. Although PAC1/c-Fos co-expression was observed across all LS subdivisions, the behavioral and endocrine effects reported here are most likely mediated by the ventral LS, which was the primary target of our microinjections based on stereotaxic cannula placement. This interpretation is further supported by evidence that ventral and dorsal LS subdivisions contribute differently to stress and affective behavior. Ventral LS circuits are strongly linked to passive coping strategies, anxiety-like responses, and modulation of hypothalamic-brainstem stress pathways, whereas dorsal LS subdivisions are more closely associated with approach-avoidance conflict, contextual threat evaluation, and the regulation of defensive behavioral states [59, 61]. The broad recruitment of PAC1 receptor-expressing neurons across these subdivisions suggests that PACAP may influence multiple, partially dissociable LS stress circuits, although the present findings primarily reflect effects mediated through ventral LS pathways.

Because the LS also regulates anxiety and fear responses [58, 59], we next examined the impact of intra-LS PACAP on anxiety-related behavior in the elevated plus-maze test, a standard assay for assessing anxiety-like behavior in rodents [76–78]. We found that local infusion of PACAP38 into the LS reduced open-arm entries and time spent in open arms, consistent with an anxiogenic-like effect, while leaving closed-arm entries and total distance traveled unchanged, thereby ruling out locomotor confounds. These results are in line with previous studies showing that intracerebroventricular PACAP or local infusions into other limbic sites (e.g., amygdala, BNST) produce similar anxiogenic-like effects in this test [39, 79–83]. Notably, intra-LS administration of the PACAP antagonist PACAP(6-38) produced the opposite effect, inducing a significant anxiolytic phenotype in the elevated plus-maze, further supporting a role for endogenous PACAP signaling in the regulation of anxiety-related behavior. Thus, our data reveal a previously unrecognized contribution of PACAP signaling within the LS in shaping stress- and anxiety-like responses, extending the neuroanatomical range of PACAP’s influence beyond traditionally implicated limbic regions. In addition to its involvement in stress and anxiety, the LS contributes to motivational processes such as self-care behavior [57, 59, 84]. Consistent with this, intra-LS PACAP38 administration reduced grooming in the sucrose splash test, an assay of self-care and motivational drive [85, 86]. Reduced grooming in this task is also observed after chronic stress exposure and can be reversed by antidepressant drugs [85, 87, 88]. Thus, PACAP-induced grooming reduction may reflect decreased motivational drive consistent with an anhedonic-like state. This interpretation is consistent with previous reports showing that intracerebroventricular PACAP38 administration disrupts motivated behaviors, producing anhedonia and reducing active social interaction [39, 89, 90].

### PACAP/PAC1 receptor pharmacology and mechanistic considerations

An important next step is to determine which receptors mediate the behavioral effects of PACAP in the LS. To directly assess the functional relevance of endogenous PACAP signaling, we examined the effects of intra-LS administration of PACAP(6-38), a widely used antagonist that blocks PAC1 and VPAC2 receptors [91, 92]. The dose was chosen based on previous intracerebral studies demonstrating effective attenuation of both endogenous and exogenous PACAP-induced behavioral effects, including anxiogenesis [82, 83, 90, 93]. Notably, PACAP(6-38) produced a robust anxiolytic effect in the elevated plus-maze, providing direct functional evidence that endogenous PACAP signaling within the LS contributes to the regulation of anxiety-related behavior. Although PACAP(6-38) does not permit definitive receptor subtype attribution, several lines of evidence strongly suggest that these effects are mediated predominantly via PAC1 receptors. First, converging anatomical data indicate that PAC1 receptors are the principal PACAP-sensitive receptor subtype in the LS (see Fig. 6; see also Allen Brain Atlas, [53, 56]), whereas VPAC2 receptor expression is negligible or absent in this region (Hannibal et al., unpublished data; see also Allen Brain Atlas, [94]). Second, from a functional neuroanatomical perspective, our data show that within the LS a substantial portion of PAC1-expressing neurons are selectively recruited under stress, as indicated by co-localization with c-Fos (see Fig. 6), supporting their functional involvement in stress-related behavior. Moreover, PACAP-positive afferents in close apposition to PAC1-expressing neurons (see Supplementary Fig. S5) provide further evidence for a preferred PAC1-mediated signaling pathway within the LS. Thus, considering both receptor distribution and the stress-activation profile of PAC1-positive neurons in the LS, the observed behavioral effects are most plausibly mediated via PAC1 receptor mechanisms.

Another key question concerns the neuronal substrates through which PACAP mediates its behavioral effects within the LS. One strong candidate involves GABAergic neurons, which constitute the predominant neuronal population in the LS [95, 96]. Supporting this idea, activation of LS GABAergic neurons has been shown to increase immobility in the forced swim test and enhance vulnerability to subthreshold chronic stress [97]. These effects appear to involve LS projections to the dorsal periaqueductal gray (dPAG), since selective activation of dPAG-projecting LS GABAergic neurons, or direct stimulation of LS-derived fibers within the dPAG, promotes passive coping and heightens susceptibility to depression-related behaviors [97]. Additional pathways may also contribute to the regulation of affective behavior by the LS. For instance, an LS-lateral hypothalamus GABAergic pathway has been identified as critically involved in modulating anxiety-related behavior in the elevated plus-maze test [98] and emotional stress-induced self-grooming [84]. Similarly, optogenetic activation of a subset of LS GABAergic neurons expressing CRF2 receptors increases stress-induced anxiety-like behavior via projections to the anterior hypothalamic area, which in turn regulates PAG- and PVN-projecting GABAergic neurons [99]. This anatomical organization raises the possibility that PAC1 receptor activation in the LS could recruit a GABA-to-GABA disinhibitory pathway (Supplementary Fig. S6) targeting e.g. PVN neurons, thereby facilitating HPA axis responses [100]. Notably, our findings indicate that PACAP38 acts locally within the LS to regulate stress-, anxiety-, and motivation-related behaviors via PAC1 receptor expressing neurons. This interpretation is supported by the observation that a subset of stress-activated (c-Fos positive) LS neurons co-express PAC1 receptors. However, it remains unclear whether these effects are mediated by a common downstream pathway or by distinct projection-specific subcircuits. This uncertainty reflects the broader complexity of LS organization, where overlapping (and in some cases functionally opposing) GABAergic subcircuits exhibit projection-dependent behavioral effects and engage in intra-septal interactions [57, 58, 61, 99]. Adding to this complexity, the LS shows marked molecular heterogeneity, with distinct gene expression profiles [101–103] and differential distributions of modulatory receptors such as PAC1. Determining the precise projection pathways through which PACAP engages LS circuitry will require projection-specific and cell-type-resolved approaches in future work.

### Limitations of the study

A limitation of the present study is that only male rats were examined. This is important because growing evidence indicates sex-dependent differences in PACAP/PAC1 receptor signaling across limbic stress circuits. For example, prior studies show that PACAP expression and function can diverge between males and females in several stress-relevant brain regions, including the hypothalamus, amygdala, and BNST, with implications for stress-, anxiety-, and trauma-related phenotypes [17, 19, 37]. In humans, a single nucleotide polymorphism in the PAC1 receptor gene (ADCYAP1R1) has repeatedly been associated with PTSD diagnosis and symptom severity particularly in trauma-exposed women [23, 27], suggesting that PAC1 receptor signaling may contribute differently to stress pathophysiology across sexes. Recent work by Velasco et al. [104] further highlights this issue by demonstrating that stress-induced PACAP upregulation in hypothalamic and extended amygdala circuits contributes to deficits in fear extinction, specifically in females. This study is particularly relevant for our findings because it underscores that limbic PACAP signaling - including in regions anatomically and functionally connected to the LS (e.g., BNST, hypothalamus, and PAG) - can exhibit female-biased plasticity after stress. These findings collectively suggest that PACAP effects within stress-regulatory circuits may be even more pronounced - or mechanistically distinct - in females. Thus, although the present study reveals robust behavioral and endocrine consequences of LS PACAP signaling in males, future work will be required to determine whether similar or potentially stronger effects occur in females. A second limitation concerns the pharmacological tools currently available for dissecting PAC1 receptor-specific mechanisms. Although our antagonist experiments were suitable to provide functional evidence for local endogenous PACAP signaling in the LS, the lack of highly potent and selective PAC1 antagonists hampers precise receptor-level attribution in other brain areas. A further limitation is that some molecular and behavioral measures were necessarily collected in separate cohorts, which limits fully establishing direct causal pathways. Thus, the development of more selective PAC1 antagonists, complementary genetic tools, and future studies employing circuit-specific, chemogenetic, or other temporally precise manipulations will be essential for identifying global brain mechanisms through which PACAP signaling regulates stress-related functions.

### Translational relevance and conclusion

Collectively, our findings highlight a nuanced, stressor-specific regulation of PACAP and PAC1 receptor expression across limbic regions, with the LS emerging as a novel and previously unrecognized site of PACAP regulation. The differential effects observed between acute and chronic stress underscore the importance of stress duration and variability in shaping PACAPergic responses in the brain. Additionally, our functional experiments further establish the LS as a critical site through which PACAP shapes stress coping, anxiety-like behavior, and motivational drive. Importantly, the demonstration that blockade of PACAP signaling within the LS produces anxiolytic effects further supports a functional role of endogenous PACAP in regulating affective behavior. Thus, these results demonstrate for the first time that heightened PACAP signaling within the LS can influence multiple affective and motivational domains relevant to stress adaptation. From a translational perspective, our data support the hypothesis that exaggerated PACAP transmission may impair adaptive stress coping and contribute to the symptom profile of certain stress- and trauma-related psychiatric disorders. This is consistent with emerging clinical evidence of elevated PACAP levels and PAC1 receptor activity in patients affected by post-traumatic stress, depression, or anxiety disorders [27–31, 33]. Elevated PACAP could thus serve as a transdiagnostic biomarker of stress-related psychiatric symptom severity, while selective pharmacological blockade of PACAP signaling represents a promising avenue for novel treatment strategies.

## Supplementary information

Suppl Mat incl Methods, Suppl Figures S1-S6,Table S1-S2

## Data Availability

Data will be made available on request.
