# Supplementary material for: Lateral septal PACAP signaling regulates stress and anxiety reactions
Source: Neuropsychopharmacology. 2026 Apr 21;51(9):1643–56. doi: 10.1038/s41386-026-02409-y (PMC13389158; doi:10.1038/s41386-026-02409-y)
Supplement: Supplementary file 1 — Suppl Mat incl Methods, Suppl Figures S1-S6,Table S1-S2 [file 41386_2026_2409_MOESM1_ESM.pdf]

Supplementary Materials

Lateral septal PACAP signaling regulates stress and anxiety reactions

Veronica Fontebasso, Federico Ferro, Magali Basille-Dugay, David Vaudry, Jens Hannibal, Nicolas Singewald, Karl Ebner*

***Correspondence:**

Karl Ebner, PhD   E-mail: karl.ebner@uibk.ac.at

This PDF file includes:

Materials and Methods

Supplementary Figures; Supplementary Fig. S1 – S6

Supplementary Tables; Table S1 - S2

Materials and Methods

Animals

Adult male Sprague-Dawley rats (250-350 g) were housed in groups of 2-4 under controlled laboratory conditions (12:12 h light/dark cycle with lights on at 07:00 AM, 22 ± 2 C°, pelleted food and water ad libitum) for at least one week after delivery from the supplier (Charles River Laboratories, Sulzfeld, Germany). All experiments were performed between 08:00-13:00 AM in strict compliance with the European Communities Council recommendations for the care and use of laboratory animals (2010/63/EU) and approved by the Austrian Animal Experimentation Ethical Board (Bundesministerium für Bildung, Wissenschaft und Forschung, Kommission für Tierversuchsangelegenheiten).

Surgery

All surgical procedures were performed under sterile conditions. Prior to surgery rats were anaesthetized with isoflurane (as inhalant: 5% induction; 2.5 % maintenance) by using a vaporizer system (EZ systems corporation, Palmer, USA). To prevent hypothermia during surgeries, animals were maintained on a heating pad. Postoperatively, rats received analgesia consisting of buprenorphine (0.1 mg/kg s.c.; three times every 8 hrs; Richter Pharma AG, Austria) and meloxicam (1 mg/kg p.o. via drinking water, Boehringer Ingelheim, Germany) and were housed individually during postsurgical recovery period. Animals were monitored daily by weighing and handling, which included removal of the dummy cannula to habituate them to the subsequent intracerebral injection procedure, thereby minimizing the stress reaction on the experimental day.

Implantation of a microinjection cannula

The stereotactic surgical procedures were performed as previously described [1,2]. Briefly, anesthetized animals were fixed in a stereotaxic frame (Stoelting, Illinois, USA) and a 23-gauge stainless steel guide cannula (15 mm length; o.d. 0.64 mm, i.d. 0.34 mm; Injecta GmbH, Germany) was implanted either unilaterally 1 mm above the right lateral ventricle (coordinates from bregma: AP 0.8 mm, ML +1.4 mm and DV -3.0 mm) or bilaterally 2 mm above the LS (coordinates from bregma: AP 0.6 mm rostral, ML +/- 1.7 mm, DV -4.0 mm, with an angle of 10°) or rostral portion of the anterolateral BNST (coordinates from bregma: AP 0.2 mm rostral, ML +/- 1.8 mm, DV -5.0 mm) by using a rat brain atlas [3]. Guide cannulas were fixed to the skull via two stainless steel screws and dental acrylic cement (Dentalon plus, Heraeus Kulzer GmbH, Germany). Thereafter, a dummy stylet was inserted into each guide cannula to prevent clogging and reduce the risk of infection. Rats were allowed a recover for 5-

7 days before experiments. The locations of the cannula track were histologically verified after experiments (see Supplementary Fig. S1).

Implantation of a jugular venous catheter

A silastic-tipped vinyl catheter was inserted into the left jugular vein of rats, routed under the skin and exteriorized at the neck of the animal as described previously [2]. The wounds were closed using metal clips. The catheter was filled with sterile saline containing gentamicin (30.000 IU/rat, Centravet, Bad Bentheim, Germany) and flushed with the same solution 2 days after surgery. On the day of the experiment, the catheter was connected to a 1-ml plastic syringe via a ~40-cm long piece of PE-50 tubing, 2 h before starting the experiment.

Behavioral procedures

Forced swim challenge

The forced swimming challenge was conducted as described previously [2]. Briefly, rats were placed individually in a square plastic tank (35 x 35 cm) filled with water (20 ± 1°C) to a depth of 30 cm. Behavior during the 5-min swim session was recorded using a video system for subsequent analysis. A trained observer, blinded to the treatment groups, scored the recordings by quantifying absolute time spent in each behavioral category. Behaviors were classified into the following three categories: (1) struggling, defined as movements during which the forelimbs broke the surface of water; (2) swimming, defined as movement of the animal induced by movements of the fore and hind limbs without breaking the water surface; and (3) floating defined as the behavior during which the animal used limb movement just to keep its equilibrium without any movement of the trunk. After the swimming session, animals were gently dried using a towel and returned to their home cages.

Elevated plus-maze test

The elevated plus maze consisted of two open arms (50 × 9 cm) arranged at right angles to two closed arms of the same size, enclosed by 30-cm-high walls. The apparatus was elevated 75 cm above the floor and placed in the center of a quiet testing room. Two lighting conditions were used depending on the experiment: PACAP38 experiments were conducted under dim red light conditions, whereas PACAP(6-38) experiments were performed under white light illumination (approximately 40-45 lux). Rats were placed individually in the center of the maze, facing one of the open arms, and allowed to explore for 5 min. Behavior was recorded using an overhead camera and analyzed with an automated

video tracking system (Videomot 2.0, TSE, Bad Homburg, Germany) by an observer blinded to experimental conditions. The following behavioral parameters were assessed: (1) entries into open arms, expressed as the ratio of open arm entries to total entries; (2) time spent on open arms, expressed as the ratio of time spent on open arms to total time spent on all arms; and (3) overall activity, including total entries into closed arms and total distance traveled. After testing, animals were returned to their home cages, and the maze was thoroughly cleaned with a neutral-odor solution between trials to eliminate olfactory cues.

Sucrose splash test

The splash test was used to assess motivational and self-care behavior. Rats were placed individually in a transparent observation cage without bedding, and a 10% sucrose solution was sprayed onto their dorsal coat. Due to its viscosity, the sucrose solution soils the fur, typically eliciting grooming behavior. Immediately after spraying, animals were behaviorally observed for 15 min. Grooming behaviors, including nose/face grooming, head washing, and body licking, were recorded and manually scored by an observer blinded to experimental group assignments. The following parameters were analyzed: (1) the total duration of grooming behavior; (2) the number of grooming bouts, defined as discrete episodes of grooming behavior, and (3) the latency of the first episode of grooming.

Stress paradigms

Acute swim stress

The forced swim challenge was conducted as described above, whereby rats were exposed to this challenge on two consecutive days [4]. Briefly, rats were exposed to two consecutive swim sessions in 20 °C water, lasting 5 min on the first day and 10 min on the second day. After each session, animals were towel-dried and returned to their home cages.

Chronic variable mild stress

Rats were subjected to a 7-day chronic variable mild stress paradigm, as previously described [4]. Each day, animals experienced one mild stressor presented in a randomized and unpredictable order, with certain stressors repeated across the week to increase unpredictability. The stressors were chosen to be mild yet effective in inducing a sustained stress response, without causing physical harm, and included commonly used procedures such as restraint, pedestal exposure, swim, and tilt-cage stress

(Table S1). Three of the stressors (swim, pedestal and restraint) were repeated during the paradigm, and all rats received restraint on the last 7th day.

Two hours after the final stress session, rats were euthanized, and their brains were rapidly extracted, snap-frozen in isopentane (Sigma-Aldrich, Austria) chilled on dry ice, and subsequently stored at -80 °C until further PCR processing.

Microinjection procedure

For bilateral microinfusions into the LS or anterolateral BNST with simultaneous blood sampling we used a rather 'stress-free' drug administration approach as described previously [2]. Briefly, stylets of guide cannulas were replaced by two 30-gauge microinjection cannulas that were 2 mm longer than the guide cannulas, thus reaching the LS or BNST. Injection cannulas were connected to a 5 cm long PE-10 tubing and filled with drug or vehicle solution. This infusion device was connected to a syringe mounted on a microinfusion pump (TSE-Systems, Bad Homburg, Germany) via a 100 cm long polyethylene tubing interconnected with a dual channel fluid swivel system (Instec Laboratories) and mounted at least 1 h for starting blood sampling. Drugs were infused over a period of 7.5 min at a defined flow rate of 0.2 µl/min (1.5 µl/injection side). After the microinjection procedure, the injection cannula was left in the guide cannula for another 2.5 min before being removed. Thereafter, animals were exposed to the forced swim challenge.

For intracerebroventricular or intraseptal microinfusions without concurrent blood sampling, rats were gently fixed inside their home cages and the stylets were carefully removed from the guide cannulas. Drugs were injected into the lateral ventricle or LS (1.5 µl/site) by manual infusion over a period of approximately 7.5 min using a 5-µl Hamilton syringe (Hamilton Instruments, Switzerland) connected via polyethylene tubing to a 30-gauge injection cannula (tip extending 1-2 mm beyond the guide). Following infusion, the internal cannula was left in place for an additional 2 min to allow for adequate drug diffusion before being slowly withdrawn. Rats were subjected to the elevated plus-maze or splash test 15 min later.

Histological verification of cannula placements

At the end of experiments, animals were sacrificed by an overdose of sodium thiopental and brains were rapidly removed, snap-frozen in 2-methylbutane solution and stored at -20°C. For histological verification of the placement of microinjection cannulas within the LS or BNST, coronal brain sections (40 µm thick) were prepared using a cryostat (Leica CM1950, Leica Microsystems, Germany). Sections were mounted onto gelatin-coated slides (Carl Roth, Germany) and stained with cresyl violet. Cannula

placements were verified under a microscope on the basis of anatomical landmarks defined in a brain atlas [3]. Only data from animals with confirmed injector tracks located within the LS (Supplementary Fig. S1) or anterolateral BNST were included in the final behavioral and neuroendocrine analyses.

Blood sampling procedure and ACTH measurements

After a 60-min habituation, the experiment started with the collection of two blood samples (0.3 ml) under basal conditions, 35 and 15 min prior to stress exposure. Bilateral infusions of drugs into the LS or anterolateral BNST started 12 min before the onset of the stressor. After injections, another blood sample was taken and 1 min later animals were exposed to the forced swim stress procedure for 5 min. During the forced swim session, behavioral output was scored and analyzed as described above. After animals were returned to their home cages three additional blood samples were collected at regular intervals (10, 30, and 60 min after the onset of the stressor). Sampled blood volumes were immediately replaced with an equal volume of heparinized saline. Blood was collected into EDTA-coated tubes containing 10 units of sodium heparin (Wako Pure Chemical Industries Ltd.), placed on ice and immediately centrifuged at 3000 g for 10 minutes at 4°C. Supernatants were collected and stored at −80°C until measurement. Plasma ACTH concentrations were determined using a commercially available immunoassay kit (MP Biomedicals, Orangeburg, NY, USA) according to the manufacturer's protocol. The intra- and inter-assay coefficients of variation were below 7 and 10 %, respectively, and a lower limit of detection of 6 pg/ml.

Drugs

PACAP38 and PACAP(6-38) (Bachem AG, Switzerland; MedChemExpress, USA) were dissolved in sterile distilled water and aliquots of stock solution (1 mg/mL) were stored at -80°C. For preparation of the working solution the liquid of concentrated stock solution was diluted with artificial cerebrospinal fluid (aCSF; 140 mM NaCl, 3.0 mM KCl, 1.25 mM $CaCl_2$, 1.0 mM $MgCl_2$, 1.2 mM Na_2HPO_4, 0.3 mM NaH_2PO_4, 3.0 mM glucose and pH adjusted to 7.4) to a final concentration of 10 or 100 µM. Vehicle animals received a 1.5 µl infusion of aCSF solution. All drugs were freshly prepared before each experiment and kept on ice during the experimental procedures.

Brain punching and quantitative real-time PCR

The method of tissue dissection, RNA extraction and quantitative reverse transcription-PCR procedure was conducted as described previously [4]. Briefly, frozen brains were placed in a cryostat chamber kept at -20°C and coronal sections (250 µm) were cut from each brain using a cryostat (Leica CM1950,

Leica Microsystems, Germany). Bilateral tissue punches from the LS, PVN, BNST, CeA, BLA and MeA were obtained using a brain punch set (Fine Science Tools, Germany), guided by the rat brain atlas [3]. Dissected brain tissue samples were collected into RNase-free Eppendorf tubes, snap-frozen on dry ice, and stored at -80 °C. Total RNA from each brain sample was extracted using the NucleoSpin RNA XS kit (Macherey-Nagel GmbH & Co. KG). For the reverse transcription, the QuantiTect Reverse Trasnscription Kit (Quiagen, USA) was performed according to the manufacturer's protocol starting with 1 µg of isolated RNA. Relative gene expression was by real-time PCR in 384-well plates using 2X Fast SYBR Green PCR Mastermix (cat. 4385612, Thermofisher), gene-specific primers (see Table S2), and cDNA samples prepared with automated liquid handling. Reactions were run on a QuantStudio Flex 12k thermal cycler (Applied Biosystems), measured in triplicate, and relative expression values were normalized to Gapdh, B2m and Actb transcript levels as reference/housekeeping genes and calculated using the $2^{-\Delta\Delta CT}$ method [5].

Immunofluorescent histochemistry

To determine the localization of PAC1 receptors (and PACAP immunoreactivity) in the LS and to identify their presence in stress-activated (c-Fos positive) neurons, immunofluorescent histochemistry was performed as described previously [2]. Two hours after swim stress exposure, animals were deeply anesthetized with sodium thiopental and transcardially perfused with 0.9% saline followed by Stefanini´s fixative (2% paraformaldehyde, 0.2% picric acid in 0.1 M phosphate buffer). Brains were removed, post-fixed overnight at 4°C, and cryoprotected in 30% sucrose solution (0.05 M PBS) for 48 h. Coronal sections (40 µm) of the LS were cut using a cryostat (Leica CM1950, Leica Microsystems, Germany) and stored in cryoprotectant at 4°C until further processing.

Immunofluorescence staining was performed as previously described [2,6]. Briefly, selected brain sections of the LS region were incubated in antigen retrieval buffer (DAKO, ChemMate, code No. S203120) diluted in distilled water (1:1. pH=6) for 90 min at 80°C (pre-heated in a microwave). Brain slices were washed with PBS/0.25% Triton X-100 (Sigma-Aldrich) and incubated in 1% hydrogen peroxide in PBS (pH=7.4) for 20 minutes to quench endogenous peroxidase activity. Thereafter, brain slices were washed with PBS/0.25% Triton X-100 (3 x 10 min/wash) and incubated in 5% normal donkey serum (Jackson ImmunoResearch) diluted in PBS/0.25% Triton X-100/bovine serum-containing albumin (Sigma-Aldrich) for a further 30 minutes at room temperature. Successively, sections were incubated in a solution containing a mouse monoclonal anti-PACAP antibody (RRID: AB_2315043, diluted 1:10) overnight at 4°C. After rinsing in PBS/0.25% Triton X-100 (3 x 10 min/wash), sections were incubated in biotinylated donkey anti-mouse antibody for 24 h at 4°C, washed and incubated in in combination with streptavidin biotin-horseradish peroxidase complex (Vector, Burlingame, CA),

biotinylated tyramide (tyramide system amplification; DuPont NEN, Boston, MA), and streptavidin-Cy2 (Amersham, Birkerød, Denmark). After blocking in 1% H_2O_2 to quench horseradish peroxidase, sections were incubated in rabbit anti-PAC1 antibody (Code 35J8, RRID: AB_2814675, diluted 1:10000) overnight at 4°C. After rinsing in PBS/0.25% Triton X-100 (3 x 10 min/wash), sections were incubated in DAKO-ENVISION system HRP (DAKO K003, Agilent Technologies, USA), diluted 1:2 in PBS/0.25% Triton X-100/bovine serum albumin (Sigma-Aldrich; USA) overnight at 4°C. On the third day, sections were washed in PBS/0.25% Triton X-100 (3 x 10 min/wash) and incubated in Alexa Fluor™ 647 Tyramide Reagent (ThernoFisher scientific, B40958) diluted 1:200 in amplification buffer (PerkinElmer, FP1052) for one hour at room temperature. For section stained for PAC1 and c-Fos only, sections were washed and incubated with a mouse anti-c-Fos (c-10) antibody (sc-271243, Santa Cruz Biotechnology, 1:500) over night and (day four), brain sections were washed and incubated with a Texas red 594 conjugated donkey anti-mouse antibody (1:200, #715-075-151, Jackson ImmunoResearch). On day five, brain sections were washed with PBS/0.25% Triton X-100 (3 x 10min/wash) and mounted on Superfrost Plus Menzel-Gläser (ThermoFisher Scientific, J1800AMNZ) in a 0.5% gelatine medium (Sigma, G2500). When the sections were dried, slides were cover slipped with DAPI (Molecular Probes, D-1306) dissolved in glycerol (Sigma-Aldrich, G9012) as a nuclear counterstain.

Photomicroscopy was performed using an iMIC confocal microscope equipped with the following objectives: X10, numerical aperture (NA) = 0.35; X20, NA = 0.75; X40, NA = 1.3. For 3D analysis images in Z-stacks photographed using the X40 objective were analyzed in IMARIS® vers. 8.4.1 (RRID: SCR_007370) from Bitplane, Switzerland (http://www.bitplane.com). Cell counting was done using the cell counter plugin in Fiji software (version 1.47q, NIH, USA) [7]. All images were adjusted for brightness and contrast either in Fiji or in Photoshop CS5 (Adobe, San Jose, CA, RRID: SCR_014199) and mounted into plates in Adobe Illustrator CS5 (Adobe).

Statistics

Statistical analysis was performed using GraphPad Prism 10 (GraphPad Software, La Jolla, USA). Details for each statistical test are marked in the figure legends. qPCR data were not normally distributed (assessed with D'Agostino-Pearson normality test) and differences between groups were analysed using nonparametric Mann-Whitney test. Behavioral data from the forced swim test with more than two groups were analyzed by one-way ANOVA followed by Dunnett's multiple comparison post hoc test. Plasma concentrations of ACTH were analyzed using a two-way ANOVA (treatment x time) with repeated measures on the last factor followed by appropriate post hoc analysis. Behavioral data from the forced swim (with two groups), the elevated plus-maze and splash test were analyzed using

Student’s two-tailed t-test, comparing drug-treated animals with respective vehicle treated controls. Data are presented as means ± SEM. Statistical significance was accepted if $p < 0.05$.

References:

1. Singewald GM, Rjabokon A, Singewald N, Ebner K. The modulatory role of the lateral septum on neuroendocrine and behavioral stress responses. Neuropsychopharmacology. 2011;36:793–804.
2. Ebner K, Fontebasso V, Ferro F, Singewald N, Hannibal J. PACAP regulates neuroendocrine and behavioral stress responses via CRF-containing neurons of the rat hypothalamic paraventricular nucleus. Neuropsychopharmacol Off Publ Am Coll Neuropsychopharmacol. 2025;50:519–530.
3. Paxinos G, Watson G. The Rat Brain in Stereotaxic Coordinates. Academic Press; 2007.
4. Ferro F, Fontebasso V, Basille-Dugay M, Vaudry D, Ebner K. Stress-type specific changes of VIP signaling in limbic regions of the rat brain. Neurosci Lett. 2025;870:138430.
5. Pfaffl MW. A new mathematical model for relative quantification in real-time RT-PCR. Nucleic Acids Res. 2001;29:e45.
6. Hannibal J, Georg B, Fahrenkrug J. PAC1- and VPAC2 receptors in light regulated behavior and physiology: Studies in single and double mutant mice. PLoS One. 2017;12:e0188166.
7. Riedel CS, Georg B, Hannibal J. Phenotyping of light-activated neurons in the mouse SCN based on the expression of FOS and EGR1. Front Physiol. 2023;14:1321007.

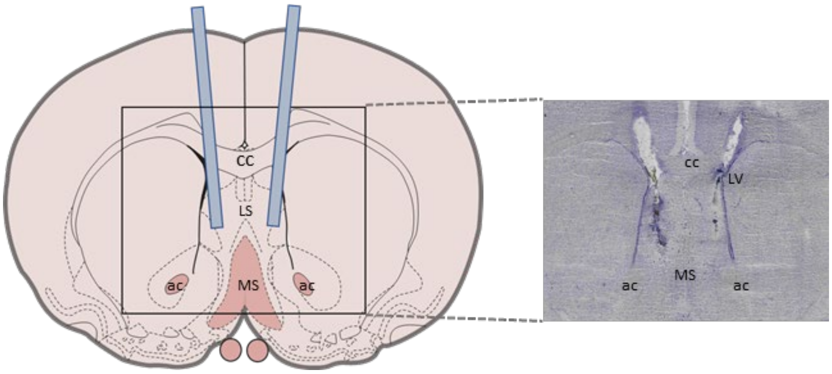

Figure S1. Injection site locations within the lateral septum (LS). Schematic drawing and representative microphotograph of a cresyl-stained coronal section of the rat brain showing the localization of microinjection cannulas for bilateral administration of vehicle or PACAP38 into the LS. Abbreviations: ac anterior commissure, cc corpus callosum, LV lateral ventricle, MS medial septum.

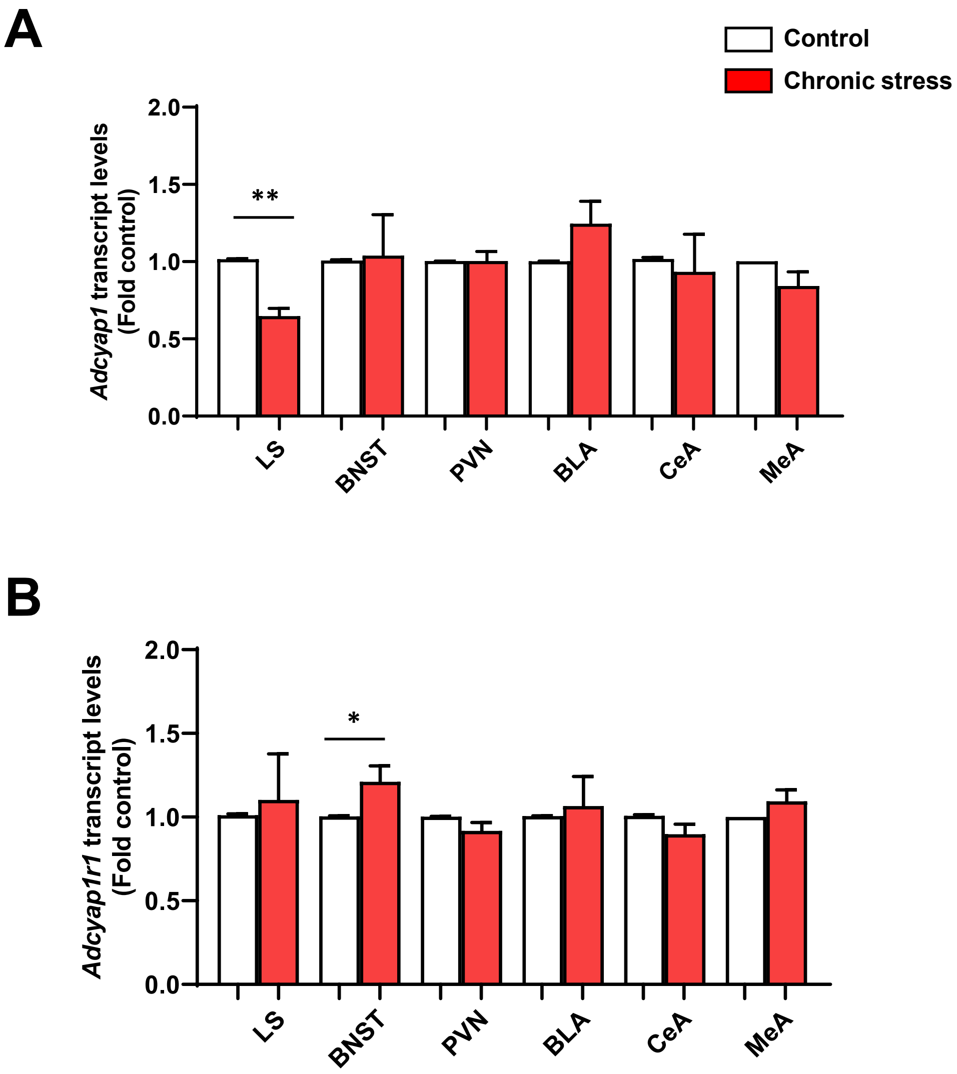

Figure S2. Effects of chronic mild stress on PACAP and PAC1 mRNA transcript levels in distinct forebrain areas of rats. Adult male rats were exposed to unpredictable chronic mild stress for seven days and distinct brain regions were harvested for quantitative PCR analyses of (**A**) PACAP (*Adcyap1*) and (**B**) PAC1 receptor (*Adcyap1r1*) mRNA transcription levels. All RNA tissue samples from each region were reverse transcribed at the same time with random hexamers to allow quantitation and normalization across samples against Gapdh, B2m and Actb transcript levels as reference/housekeeping genes. Fold changes in gene expression of chronic stressed animals (red bars, n=5-6) were calculated using the $2^{-\Delta\Delta Ct}$ method and compared to unstressed controls (white bars, n=4). Abbreviations: LS lateral septum, BNST bed nucleus of the stria terminalis, PVN paraventricular nucleus, BLA basolateral amygdala, CeA central amygdala, MeA medial amygdala. Data are expressed as group means ± SEM. * $p < 0.05$, **$p < 0.001$ compared to controls (Mann-Whitney U-test).

A

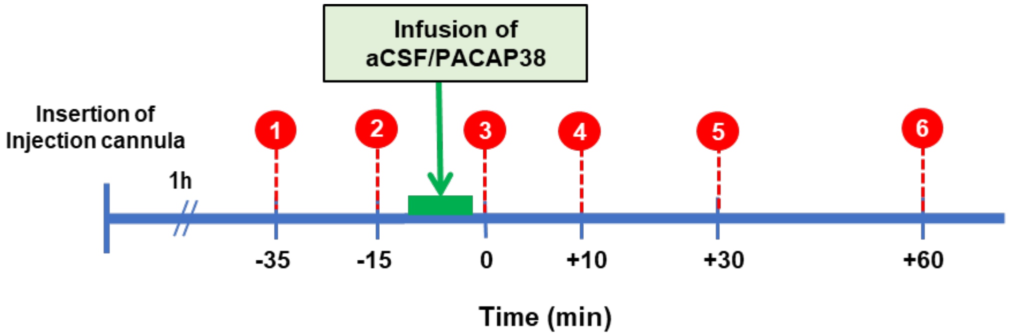

B

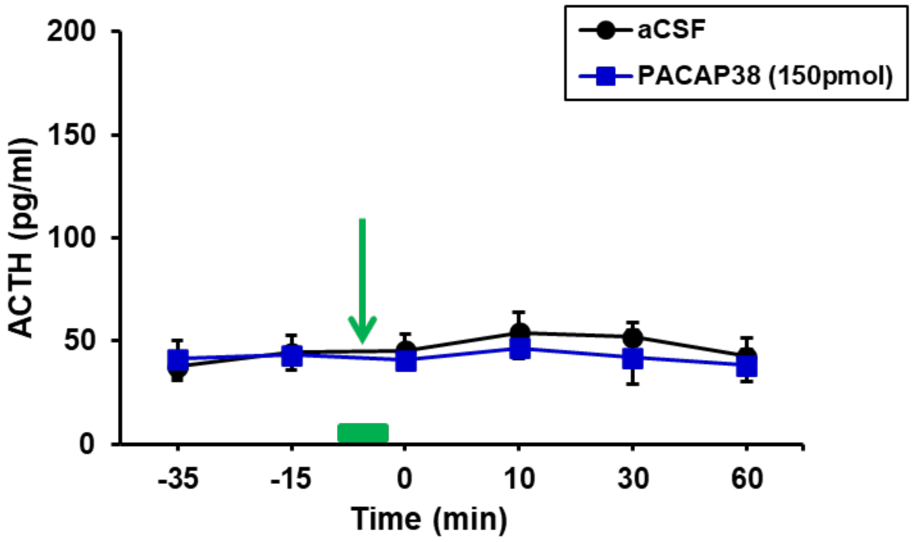

Figure S3. Effects of PACAP38 administration into the LS on basal ACTH levels. (**A**) Schematic illustration of the experimental design with the timeline of blood sampling (red circles), and drug infusion (green bar). Experiment started with insertion of the infusion device (bilateral injection cannulas connected to a microinfusion-pump) at least 1 h before blood sampling started. Drugs were infused automatically at a constant flow rate over a period of 7.5 min without any stressful manipulations (e.g. such as capturing or restraining animals) before and during the infusion procedure. Blood samples were collected at regular intervals before (-35 and -15 min) and after drug infusion (10, 30, and 60 min). (**B**) Time course of ACTH plasma levels before and after PACAP38 administration. Note that ACTH levels did not differ between groups. The green bar/arrow indicates timing of intra-LS infusion. Data are expressed as mean ±SEM, n=3-4 animals per group.

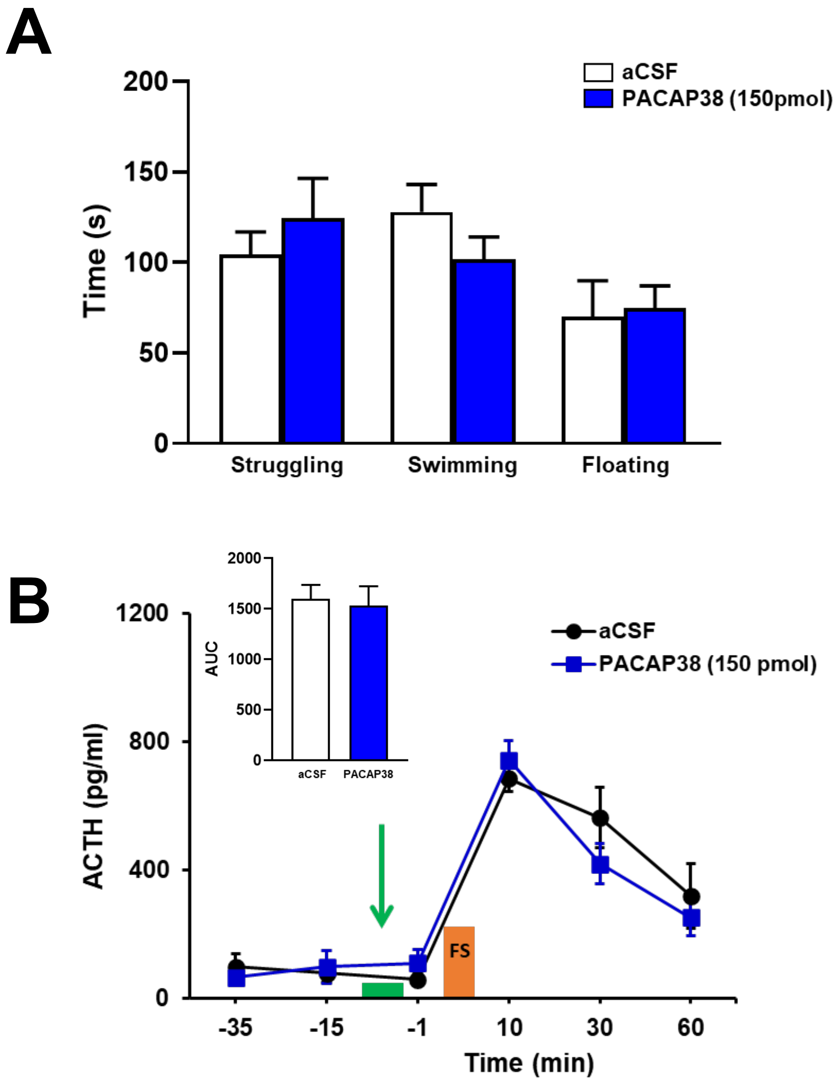

Figure S4. Effects of bilateral microinjection of PACAP38 into the rostral portion of anterolateral BNST on behavioral and neuroendocrine responses to forced swim stress. (**A**) Intra-BNST injection of PACAP38 (150 pmol/site) showed no significant alterations in behavioral stress coping during a 5-min forced swim session compared to vehicle-injected control animals. Data are expressed as mean ± SEM, n=6-10 per group. Student's t-test unpaired. (**B**) Swim stress induced an increase of plasma ACTH levels in both groups. Administration of PACAP38 into the BNST had no significant effect on ACTH plasma levels, as neither basal nor stress-induced ACTH levels differed between PACAP38 treated rats and aCSF-injected controls. The inset shows the area under the curve (AUC) quantification of ACTH levels, with no significant differences between groups. The green arrow/bar indicates timing of intra-BNST infusion, the orange bar the forced swim (FS) stress exposure. Data are expressed as mean ± SEM, n=5-7 per group. Two-way ANOVA, with post hoc Bonferroni´s multiple comparison test.

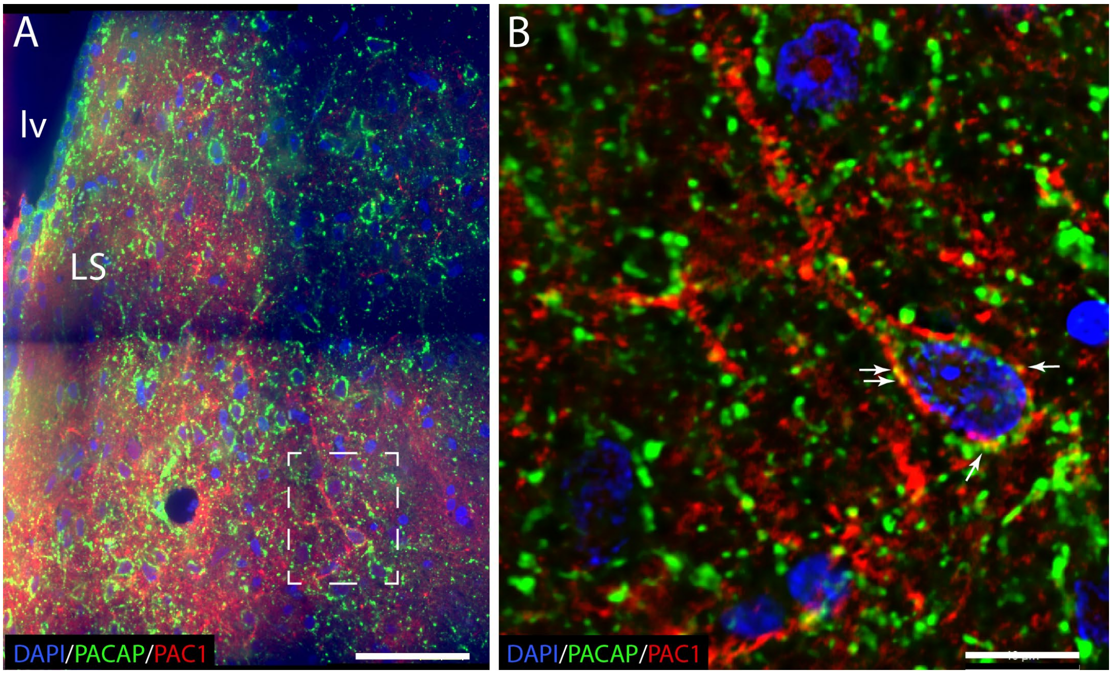

Figure S5. Lateral septum of a normal (unstressed) rat stained for PACAP (green), PACAP type 1 (PAC1) receptor (red) and DAPI (blue) using a mouse monoclonal anti-PACAP antibody (RRID:AB_2315043) and a rabbit anti-PAC1 antibody (RRID:AB_2814675). Confocal microscopy revealed PACAP-positive nerve fibers innervating lateral septal neurons (**A**), which express PAC1 receptors along their cell membranes and processes. In a higher magnification image (**B**), arrows indicate examples of PACAP-positive nerve terminals positioned in close apposition to PAC1 immunoreactivity on the cell membrane, likely representing synaptic contacts. Scale bars: A, 40 µm, B, 10 µm. LS, lateral septum; lv, lateral ventricle.

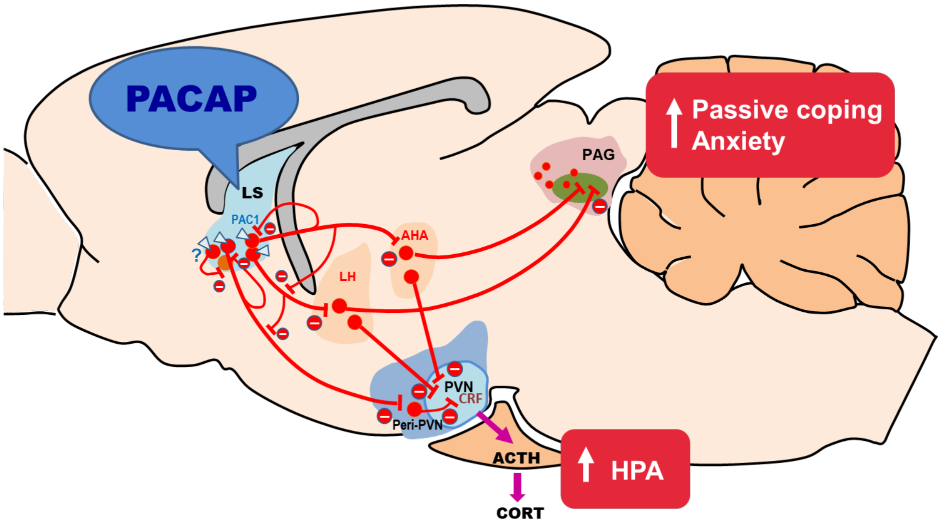

Figure S6. Proposed model how increased LS PACAP signaling promotes anxiety-like behaviors, passive stress coping and enhances HPA stress responses. We hypothesize that enhanced activity of PAC1-expressing GABA neurons, particularly in the ventral LS, which preferentially project to hypothalamic areas (e.g. AHA, LH or peri-PVN), regulates PAG- and PVN-projecting GABAergic neurons. This leads to disinhibition of stress- and anxiety-regulating target cells (e.g. CRF neurons in the PVN). Through this PACAP-mediated engagement of a GABA-to-GABA disinhibitory pathway, septal PACAP may facilitate increases in stress hormone release as well as anxious and defensive behaviors (e.g immobility in the forced swim challenge). Previous reports also suggest that LS neurons exhibit robust self-activity-dependent inhibition due to extensive recurrent collaterals and lateral inhibition; this is illustrated outside the LS in the schematic, but exists preferentially between LS intrinsic circuits (Besnard & Leroy,2022). Blue triangles indicate PAC1 receptors; red circles represent GABAergic neurons; red lines denote axons of inhibitory GABAergic neurons. Abbreviations: AHA anterior hypothalamic area, LH lateral hypothalamus, LS lateral septum, peri-PVN perinuclear region of the PVN, PAG periaqueductal gray, PVN paraventricular nucleus of the hypothalamus.

Table S1. Stressors used in the chronic variable mild stress paradigm. [a]

Day	Stressor	Duration (min)
Day 1	Restraint stress	60
Day 2	Swim stress	10
Day 3	Pedestal stress	30
Day 4	Tilt cage 45° stress	180
Day 5	Pedestal stress	30
Day 6	Swim stress	10
Day 7	Restraint stress	60

[a] Rats were exposed to a seven days unpredictable variable mild stress paradigm in which a single stressor was presented each day.

Table S2. The sequence of primers used in RT-qPCR analysis.

Gene	Forward primer	Reverse primer
Adcyap1	CACGCTCCCTCCTAGTTTTGTG	GGACTTGTTTGCCGAAGCTAA
Adcyap1r1	GGTCTTGCTCTATGAAACCTTAAAGG	CCATACTCTGAGGACCACATCCT
B2m	TCTTTCTACATCCTGGCTCACACT	ACGGTTTTGGGCTCCTTCA
Gapdh	CAGCCTCGTCTCATAGACAAGATG	CAATGTCCACTTTGTCACAAGAGAA
Actbf	ACCGTGAAAAGATGACCCAGAT	CACAGCCTGGATGGCTACGTA
